# Transcriptional regulation drives heteroresistance in *Pseudomonas aeruginosa*: evidence from genomic and targeted gene expression analysis

**DOI:** 10.1128/msystems.00538-26

**Published:** 2026-08-18

**Authors:** Weijie Feng, Jia Hou, Gang Li, He Yin, Tiantian Dang, Guangqi Li, Jiaming Li, Min Li, Chunhua Song, Sean X. Leng, Zhijun Zhao

**Affiliations:** 1 General Hospital of Ningxia Medical University 74747, Yinchuan, Ningxia, China; 2 Department of Respiratory and Critical Care Medicine, General Hospital of Ningxia Medical University, Yinchuan, Ningxia, China; 3 Medical Laboratory Center, General Hospital of Ningxia Medical University, Yinchuan, Ningxia, China; 4 Central Laboratory, Peking University First Hospital Ningxia Women and Children’s Hospital (Ningxia Hui Autonomous Region Maternal and Child Health Hospital), Yinchuan, Ningxia, China; 5 Division of Hematology, The Ohio State University Wexner Medical Center, The James Cancer Hospital 12306, Columbus, Ohio, USA; 6 Johns Hopkins Center on Aging and Immune Remodeling, Division of Geriatric Medicine and Gerontology, Department of Medicine, Johns Hopkins University School of Medicine and Bloomberg School of Public Health, Baltimore, Maryland, USA; 7 Third Clinical Medical College, Ningxia Medical University 105002, Yinchuan, Ningxia, China; Child Health Research Foundation, Dhaka, Bangladesh

**Keywords:** heteroresistance, *Pseudomonas aeruginosa*, resistance mechanisms, transcriptional regulation, two-component system

## Abstract

Bacterial heteroresistance, characterized by the presence of antibiotic-resistant subpopulations within phenotypically susceptible bacterial colonies, poses significant challenges to clinical antimicrobial susceptibility testing and treatment outcomes. While heteroresistance has been increasingly recognized across diverse bacterial species, the underlying mechanisms in *Pseudomonas aeruginosa* remain incompletely understood, particularly regarding carbapenem and monobactam resistance. In this study, we employed a multi-omics approach that integrates phenotypic screening, whole-genome sequencing, core genome multilocus sequence typing, and quantitative transcriptomic analysis to systematically investigate the phenotypic characteristics and mechanistic basis of heteroresistance in 38 non-duplicative clinical *Pseudomonas aeruginosa* samples. The findings reveal a high prevalence of heteroresistance among clinical isolates, and it is difficult to detect using currently commonly used automated detection methods, such as the VITEK 2 system. The comparative genomic analysis demonstrated that resistant and non-resistant subpopulations exhibit identical resistance gene repertoires, with no detectable genetic mutations, gene amplification, or plasmid acquisition. Instead, we identify differential expression of key resistance genes (*mexA*, *ampC,* and *oprD*) as the primary driver of heteroresistance, with the two-component system ParS/ParR negatively correlating with *oprD* expression (*r* = −0.797, *P* < 0.05). Furthermore, phenotypic validation using efflux pump and AmpC inhibitors substantiated the functional contribution of these mechanisms. Our findings indicate that *Pseudomonas aeruginosa* utilizes dynamic transcriptional regulation rather than genetic alteration to evade antibiotic pressure, representing a distinct evolutionary strategy for survival under antimicrobial stress.

**IMPORTANCE:**

The clinical implications of our findings are profound. The stable inheritance of HR phenotypes implies that resistant subpopulations can persist as cryptic reservoirs within susceptible populations, evading routine antimicrobial susceptibility testing and potentially leading to relapse or treatment failure. Moreover, the fact that HR is driven by transcriptional rather than genetic mechanisms means that standard molecular diagnostics targeting resistance genes would fail to identify these strains. This highlights the urgent need for diagnostic approaches that capture functional resistance phenotypes or transcriptional signatures.

## INTRODUCTION

The rising tide of antimicrobial resistance represents one of the most pressing challenges to global public health. Heteroresistance (HR) is a phenomenon where a subpopulation of isogenic bacterial cells exhibits elevated antibiotic resistance compared to the majority susceptible population, and it has emerged as a stealthy contributor to therapeutic failure and diagnostic inaccuracy (1). The antimicrobial HR is hindered by a lack of standardized definitions and detection methods, which obscures its true clinical prevalence and mechanistic basis (2), precludes a consensus on its clinical significance, and hampers the development of effective countermeasures.

*Pseudomonas aeruginosa* (PA), a common opportunistic pathogen (3), is not only intrinsically resistant to multiple antibiotics but also highly prone to developing drug resistance, leading to challenging multidrug-resistant infections (4, 5). The sporadic reports have indicated the presence of HR in PA (6, 7), while systematic research on heteroresistant *Pseudomonas aeruginosa* (HR-PA) remains insufficient; particularly, so far, there has still been no answer to the key question of whether HR-PA is driven by genetic mutations or stems from transcriptional regulation of resistance genes.

Most previous studies on HR in gram-negative bacteria have focused on genetic alterations, such as gene amplification, single-nucleotide polymorphisms (SNPs), or the acquisition of mobile genetic elements (8–10). However, the role of transcriptional regulation in mediating HR, especially in PA, is less explored. Also, the genetic relatedness of resistant and susceptible subpopulations is not always rigorously confirmed in HR studies, potentially leading to confounding interpretations.

Here, we present evidence for a high prevalence of HR among 38 clinical isolates of PA tested against nine antibiotics. We demonstrate that PA mediates HR to carbapenems and monobactams through stable regulation of innate drug resistance gene expression. Crucially, this differential gene expression may not be mediated by known regulatory mechanisms. The phenotypic validation suggests that the two-component system (TCS) may underlie differential gene expression within bacterial colonies. This study elucidates the molecular mechanisms underlying HR in PA and highlights the challenges posed by emerging bacterial resistance mechanisms to current clinical laboratory diagnostics, while emphasizing the importance of addressing this emerging bacterial resistance phenomenon.

## RESULTS

### Phenotypic characteristics of HR-PA and their impacts on clinical laboratory diagnosis

**Fig 1:**
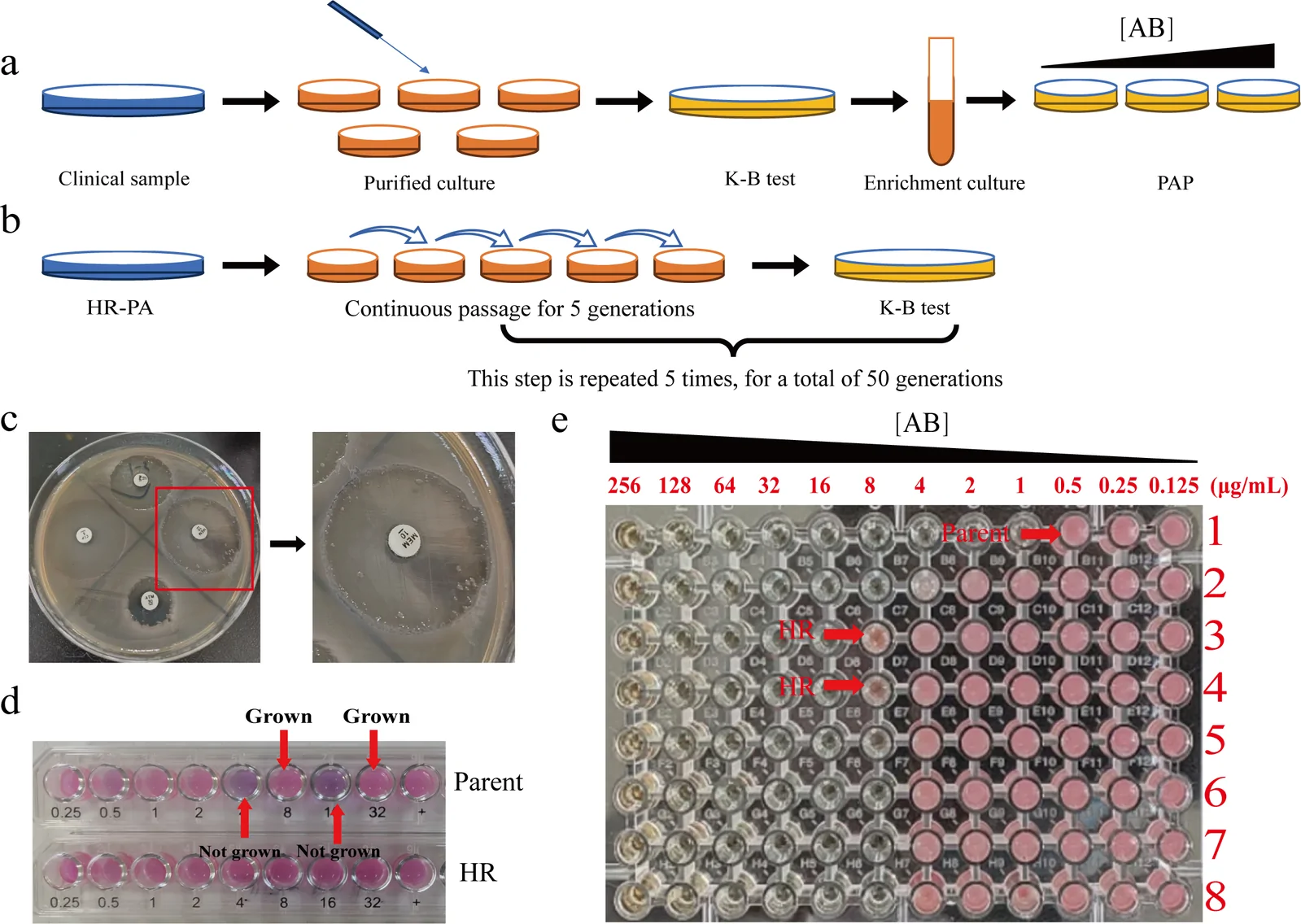
Characteristics of phenotypic HR-PA in this study. (**a, b**) HR-PA schematic diagram of screening scheme and genetic stability scheme. Subpopulations exhibiting the highest fold differences in MIC were selected for subsequent mechanistic studies.(**c**) Positive initial screening phenotype for HR, showing satellite colonies (indicated by arrows) growing within the inhibition zone of a disk diffusion susceptibility test. (**d**) Isolates with HR occasionally exhibit discontinuous growth patterns, as shown in the figure, on broth microdilution susceptibility plates. This phenomenon is referred to as “skip well.” (**e**) Phenotypic characteristics of HR in broth dilution. According to the definition of HR, a resistant subpopulation is confirmed when its MIC is atleast eightfold higher than that of the main susceptible population (parental strain). Taking the PAP of meropenem HR patterns in PA32 as an example, the subpopulations in wells 3 and 4 exhibit an MIC of 8 µg/mL, which is 16-fold higher than the parental MIC (0.5 µg/mL), confirming them as HR-positive. PAP, population analysis profiling; HR-PA, heteroresistant *Pseudomonas aeruginosa;* [AB], antibiotic concentration.

Regarding the definition of HR, we refer to the viewpoint of Herve Nicoloff and colleagues (9), which states that the resistance level of the resistant subpopulation (usually expressed in MIC) exceeds that of the dominant population by more than eightfold. Based on this definition, we performed HR testing for nine different antibiotics on 190 isolates obtained from 38 clinical samples of PA. These antibiotics were all recommended clinical surveillance agents for PA by CLSI (the method summary is shown in Fig. 1a, and detailed methods are presented in Materials and Methods). Previous studies have reported HR of PA isolates against some antibiotics used in our study (e.g., carbapenems and quinolones), but no detailed reports have been published for the remaining antibiotics.

Finally, we confirmed that 3.22% (55/1710) of the 1,710 bacterial-antibiotic combinations exhibited HR (Table 1). In the Kirby–Bauer disk diffusion susceptibility test (K-B test) initially showing potential HR, population analysis profile (PAP) test revealed 34.52% (28/84) false positives. To evaluate the efficacy of broth dilution as a confirmatory HR test, we performed MIC determinations using broth dilution on 84 bacterial-antibiotic combinations suspected of HR. Based on HR definitions, 71 isolates met the criteria (Table S1). Compared to the PAP test, the broth microdilution method yielded 29.09% (16/55) false positives. To investigate the impact of HR-PA in our hospital on clinical testing, we compared the confirmed experimental results with those reported by the VITEK 2 system. The results showed that only four bacterial-antibiotic combinations were identified as “resistant” by the VITEK 2 system (Table 1). In summary, our screening study identified 21 cases of HR in 38 clinical samples. Among the nine tested antibiotics, HR incidence rates ranked from highest to lowest as follows: MEM, 25.45% (14/55); ATM, 20% (11/55); LVX, 18.18% (10/55); IPM, 16.36% (9/55); AMK, 14.54% (8/55); CZA, 3.64% (2/55); TOM, 1.82% (1/55); CIP, 0% (0/55); and PMB, 0% (0/55). Previous reports have documented LEV of HR in PA. Based on this, we focused on isolates exhibiting simultaneous HR to MEM, IPM, and ATM.

**TABLE 1:** Confirmation of heterogeneous drug resistance in *Pseudomonas aeruginosa*

Drug sample	PAP^*a*^							VITEK 2^*b*^						
	MEM	IPM	LVX	TOB	ATM	AMK	CZA	MEM	IPM	LEV	TOB	ATM	AMK	CZA
2	8/0.5	16/0.5	16/0.5					≤0.25	1	0.5				
8	8/0.25		4/0.5			16/1		≤0.25		1			≤2	
14				2/0.25		8/0.5					≤1		≤2	
15						16/1							≤2	2
32	8/0.5		16/0.5		≥64/8	16/1		0.5		0.5		16	≤2	
35	4/0.5	8/0.5				1/0.12		0.5	2				≤2	
43					32/0.5							**32**		
44	16/1	16/2			32/0.5			2	1			4		
47		32/2							**8**					
50	≥64/8	≥64/8				8/0.5	≥64/2	**16**	**16**				≤2	2
52			2/0.25		16/2					0.5		2		
53	32/0.5		8/0.5					1		1				
54	16/1	32/1	4/0.5		16/2			1	2	0.5		2		
74	16/0.5				16/1		4/0.25	0.5				4		2
75	≥64/1		2/0.12					1		0.25				
76			4/0.25		32/1					1		1		
79	32/0.5	8/1			32/2			≤0.25	2			16		
86	32/0.5	16/0.5	2/0.25		≥64/4			≤0.25	2	0.25		16		
88	4/0.5				≥64/2	2/0.25		≤0.25				2	≤2	
89		32/1				8/1			2				≤2	
102	8/0.25		1/0.12		4/0.5			≤0.25		≤0.12		4		

^*a*^ Values indicate resistance subpopulation MIC/non-resistant subpopulation MIC.

^*b*^ Bold values indicate HR-PA correctly identified by VITEK 2.

**Fig 2:**
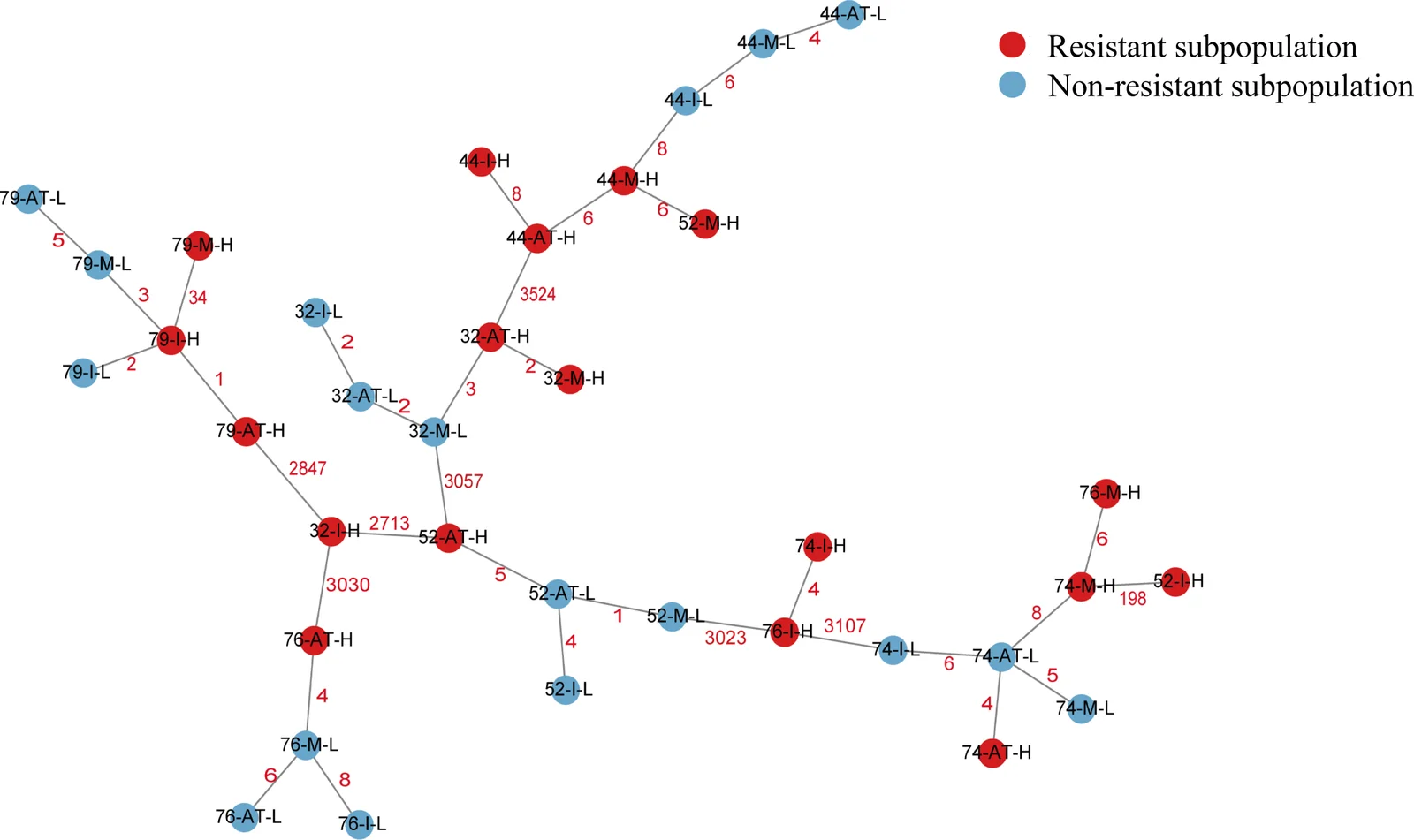
PA heteroresistance cgMLST analysis. The cgMLST typing results are presented in the form of a minimum spanning tree. Nodes in red and blue represent resistant subpopulations and non-resistant subpopulations, respectively, whereas the numbers between nodes indicate genetic distances between samples. Subpopulations with genetic distances less than 10 were considered to exhibit homology. It is noteworthy that the genetic distance between 79-I-H and 79-M-H exceeds 10; however, since both strains originate from the same parental strain, 79-M-H was not excluded. AT, ATM; M, MEM; I, IPM.

The broth microdilution method results demonstrated that PA32, PA44, PA52, PA74, PA76, and PA79 exhibited HR to MEM, IPM, and ATM simultaneously. To further investigate the genetic feasibility of using the broth microdilution method as an alternative to PAP for HR confirmation tests, we performed whole-genome sequencing on resistant and non-resistant subpopulation isolates detected in the six sample cases (detailed methods are presented in Materials and Methods). Sequencing results were utilized for core genome multilocus sequence typing (cgMLST), and a whole-genome phylogenetic tree was constructed for validation. The minimum spanning tree generated by cgMLST and the phylogenetic tree constructed from whole-genome sequencing revealed significant homology between the resistant and non-resistant subpopulation of PA44 and PA79; homology was observed only between PA74 and PA32 in ATM and MEM groups, while homology was detected exclusively between PA76 and PA52 in the ATM group. All six sample cases exhibited high homology among non-resistant subpopulations, with strains showing abnormal homology predominantly concentrated in resistant subgroups (Fig. 2; Fig. S1). In subsequent mechanism studies, samples with greater genetic distance were excluded, and remaining samples were subjected to further research.

It is noteworthy that cgMLST based on the highly conserved core genome of bacterial chromosomes enables the identification of core genes with inconsistent sequences. To rule out core genome mutations as potential driving factors for HR in PA, we analyzed the annotation results of the cgMLST data. By performing core genome sequence analysis on genetically related subpopulations, we identified significant differences in the PA0001 gene between resistant and non-resistant subpopulations (Table S2). Through NCBI search, this gene was identified as the *dnaA*. This gene encodes the most conserved DNA replication initiation protein in bacteria, which not only initiates chromosomal replication but also functions as a transcription factor acting as either an activator or repressor. Using SnapGene software, we translated the divergent *dnaA* gene sequences and performed amino acid sequence analysis of the encoded products against the amino acid sequence of the PAO1 (ID: NC_002516.2) *dnaA* obtained from the NCBI database. Finally, we found no significant mutations in other samples except for a point mutation (P141A) in the *dnaA*-encoding products of the meropenem- and aztreonam-resistant subpopulations in PA44 (Fig. S2).

### Resistance is stable and lasting for the HR isolates included in this study

The HR of clinically common gram-negative bacteria (such as *Escherichia coli*, *Klebsiella pneumoniae,* and *Acinetobacter baumannii*) has been found to be transient and unstable in previous reports (9–11). This phenotype is primarily caused by genetic mechanisms, including insertional mutations, copy number variations of resistance genes, transposons carrying resistance genes, or plasmids harboring high-copy resistant genes. To evaluate the stability of HR-PA, we subjected the resistance subpopulations included in this study to serial passage for 50 generations on antibiotic-free media and tested changes in their resistance phenotypes (the method summary is shown in Fig. 1b, and detailed methods are presented in Materials and Methods). The results demonstrated that the resistance levels to the corresponding antibiotics remained stable in all 12 resistant clones throughout the entire passage cycle (Table S3).

**Fig 3:**
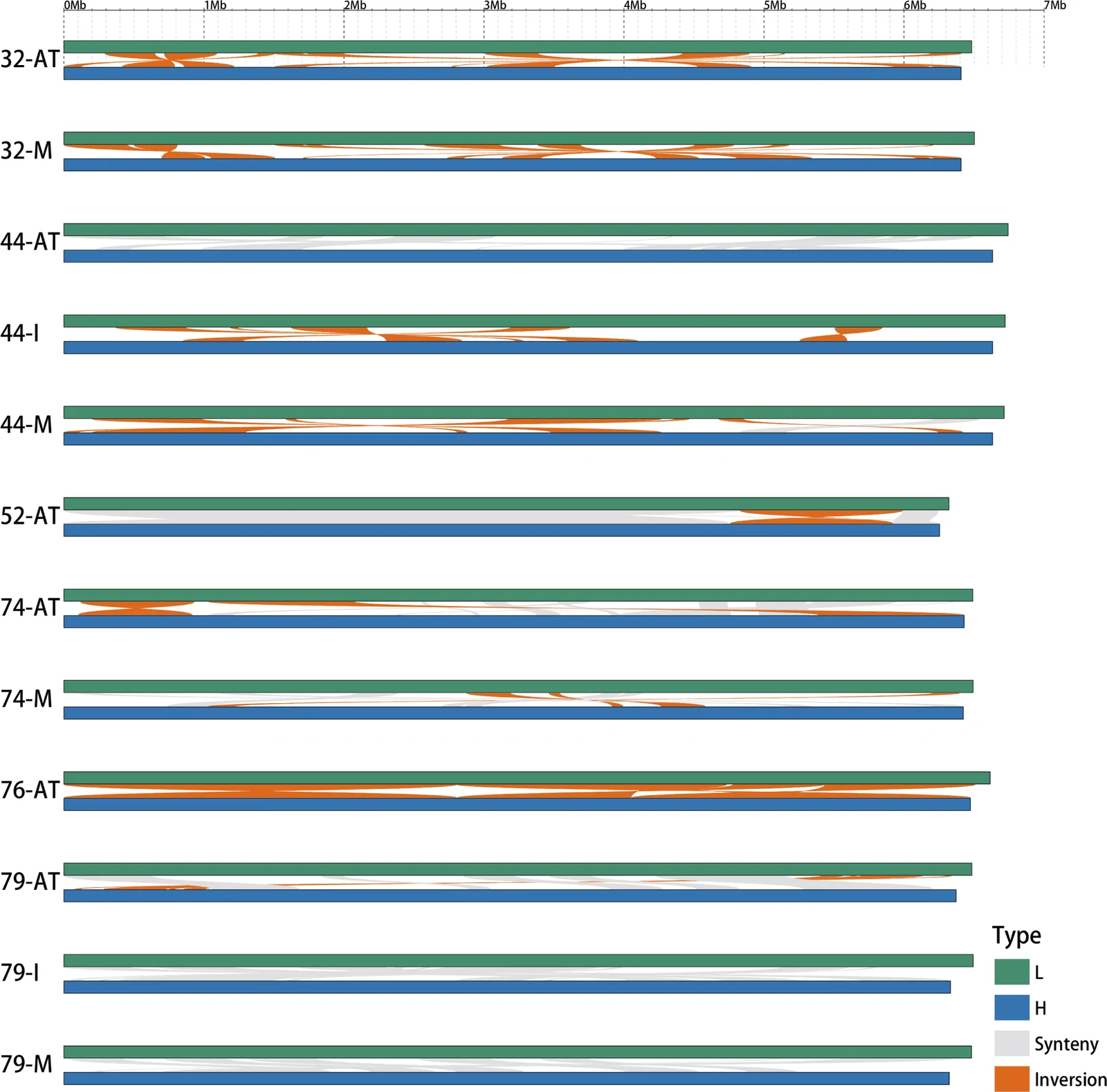
Chromosome collinearity analysis to examine the genomes of each subgroup. Since our non-resistant subgroup was only sequenced by Illumina, it was affected by gene assembly, resulting in severe gene inversions in 32-ATM, 32-MEM, 44-IPM, 44-MEM, 52-ATM, 74-ATM, and 76-ATM. AT, ATM; M, MEM; I, IPM.

To elucidate the genetic mechanisms behind stable HR phenotypes, we analyzed whole-genome sequencing (WGS) data from 12 resistant subpopulation clones and their corresponding non-resistant clones included in the study. The results revealed the absence of the previously identified unstable genetic mechanisms in these strains. Core genome sequences exhibit high homology between resistant and non-resistant subpopulations (see above). To further investigate this, we conducted chromosomal collinearity analysis, which conclusively confirmed the absence of unstable genetic mechanisms such as insertional mutations or transposons between the resistant and non-resistant subpopulations (Fig. 3).

### Stable HR is associated with abnormal expression of stable resistance genes

**Fig 4:**
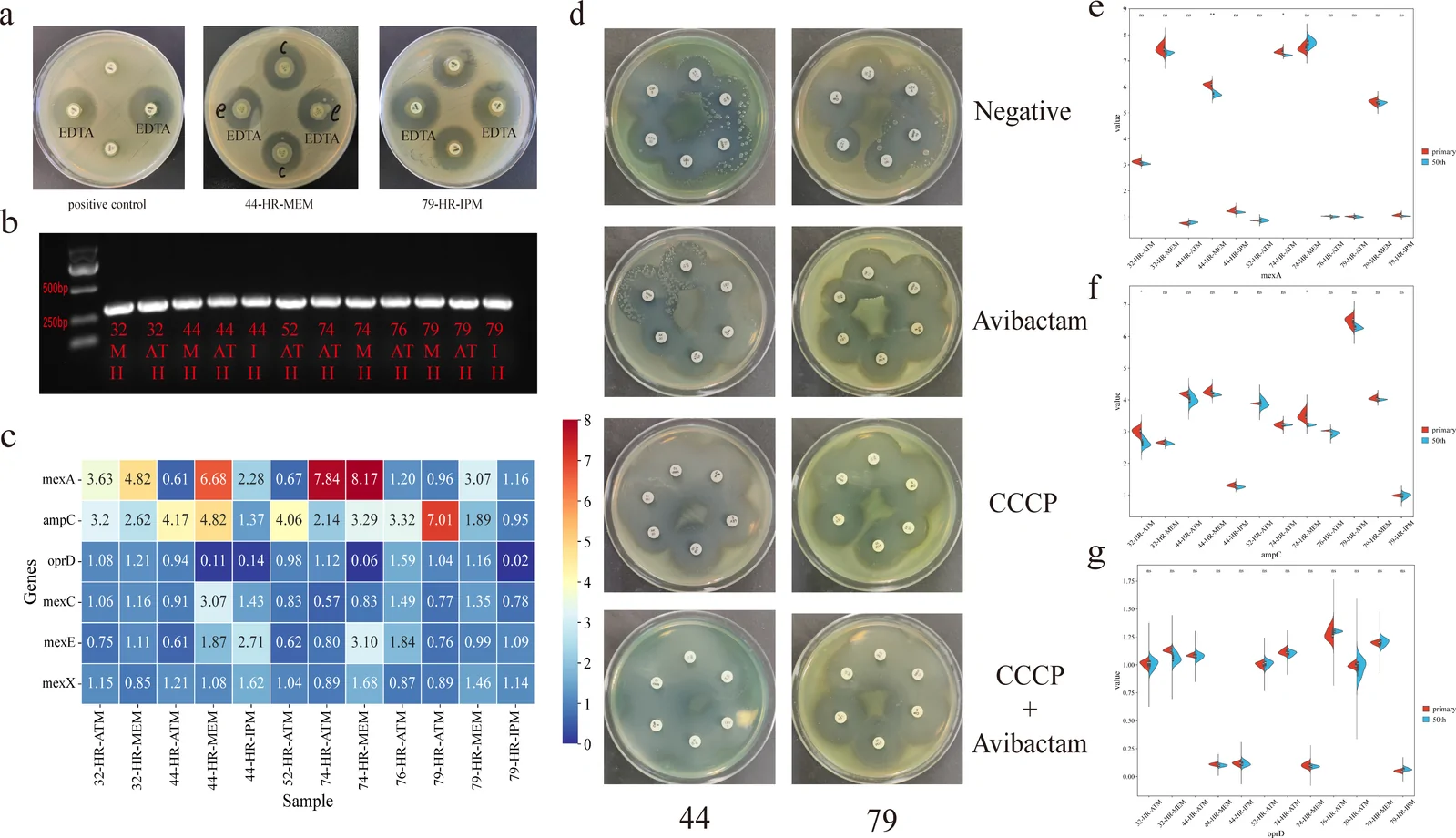
Stable HR is associated with abnormal expression of stable resistance genes. (**a, b**) We validated the reliability of our drug resistance gene annotation results through phenotypic and genotypic validation. Full-length gels are presented in Fig. S3. (**c**) Data within the heatmap represent the relative expression levels in resistant subpopulations. (**d**) The role of the efflux pump MexAB-OprM and β-lactamase AmpC in HR-PA. (**e to g**) The expression levels of resistant genes remained relatively stable. After 50 passages without antibiotic pressure, the expression levels of resistant genes in the resistant subpopulations were stable. Notably, there were differences in *ampC* gene expression between 32-HR-ATM and 74-HR-MEM isolates (*P* < 0.05), but no significant phenotypic changes were observed. Moreover, the expression levels of these genes remained statistically significant (relative gene expression level > 2) after 50 passages. Therefore, we still consider their expression to be stable. AT, ATM; M, MEM; I, IPM; CCCP, carbonyl cyanide m-chlorophenyl hydrazone. *: *P* < 0.05; **: *P* < 0.01; ns, not significant.

The formidable resistance of PA is due to its genome’s various natural resistance genes, such as the resistance-nodulation-division (RND)-type efflux pumps and β-lactamases (12, 13). The Comprehensive Antibiotic Resistance Database (CARD) annotation for resistance genes from the WGS data indicates no differences in gene composition between resistant and non-resistant subpopulations (Table S4). Further, we examined whether the MEM-resistant subpopulation and the IPM-resistant subpopulation contained carbapenemase by eCIM/mCIM analysis. Results revealed that no carbapenemases were detected in the tested HR subpopulation (Fig. 4a). Then, we confirmed the CARD annotation through PCR detection of the OXA-50 gene. Results showed the presence of the OXA-50 gene across all cases (Fig. 4b), which demonstrated the accuracy of the CARD annotation.

The next question is how does PA generate HR if the whole-genome sequencing data did not show the differences in resistance genes? It has been reported that resistance to carbapenems and monobactams in PA is associated with the AmpC enzyme, the multidrug efflux pump, and the porin protein OprD (14). To verify whether HR phenotype results from the expression of the aforementioned resistance-related genes, we isolated the total RNA from the 12 paired resistant and non-resistant subpopulations and examined the expression levels of *ampC*, *mexA*, *mexC*, *mexE*, *mexX*, and *oprD* in the isolates. First, we confirmed the antimicrobial susceptibility phenotypes of the resistant and non-resistant subpopulations using the K-B test (Table S5). Then, the qRT-PCR results showed elevated *mexA* expression in meropenem-resistant strains (*P* = 0.0025), enhanced *ampC* expression exclusively in the aztreonam-resistant strains (*P* = 0.0091), and downregulated *oprD* expression solely in the imipenem-resistant strains (*P* = 0.004), while the strain showing resistance to all three drugs exhibited aberrant expression of *mexA*, *ampC*, and *oprD* (Fig. 4c).

Next, to further validate the role of the drug-resistant genes on the HR in PA, we performed the rescue assay by inhibiting the function of *mexA* and *ampC* using avibactam as an AmpC enzyme inhibitor and carbonyl cyanide m-chlorophenyl hydrazone (CCCP) as an efflux pump inhibitor. We selected two parental isolates exhibiting HR to both carbapenem and aztreonam to observe the effects of the two inhibitors on satellite-like colonies within the inhibition zones on the plates. The results showed that CCCP effectively inhibited the growth of satellite-like colonies within the carbapenem inhibition zone, but its effect was not particularly significant against aztreonam; avibactam effectively inhibited the growth of satellite-like colonies within the aztreonam inhibition zone, but had minimal efficacy against carbapenems. On plates containing both CCCP and avibactam, no satellite-like colonies were observed within the inhibition zones of either carbapenems or aztreonam (Fig. 4d). These results demonstrated that HR in PA is dependent on the expression of these drug-resistant genes.

The above results revealed that HR-PA may be driven by abnormal expression of resistant genes, with its resistance level remaining unaffected even in antibiotic-free environments. To further validate the stability of aberrantly expressed resistant genes in HR-PA, we compared the expression levels of resistant genes in HR clone isolates after 50 generations with those of initial HR clones. The results demonstrated no significant changes in resistant gene expression across isolates, confirming that stable HR is maintained through differential expression of stable resistant genes (Fig. 4e through g).

### Potential driving mechanisms of differential expression of resistant genes in HR

**Fig 5:**
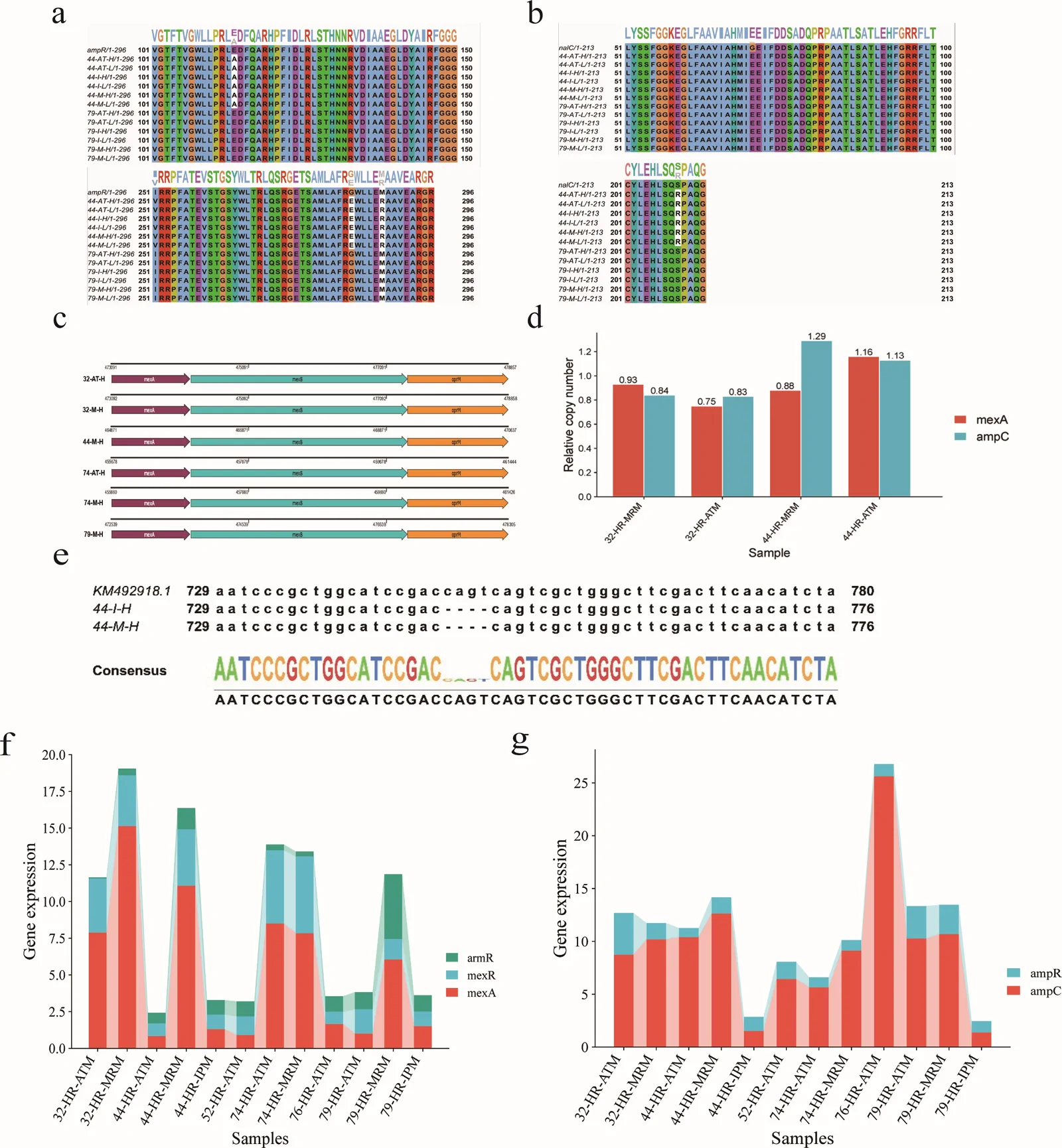
Genome of HR-PA. (**a, b**) The regulatory genes *ampR* and *nalC* of AmpC and MexAB-OprM were visualized in six sample pairs. The *ampR* point mutation patterns in 44-AT-H, 44-AT-L, 44-M-H, 44-M-L, 44-I-H, and 44-I-L exhibited *ampR* point mutation patterns of E114A, G283E, and M288R; *nalC* point mutations were G71E and S209R. In the 79-AT-H, 79-AT-L, 79-M-H, 79-M-L, 79-I-H, and 79-I-L samples, no point mutations were detected in *ampR*; the point mutation pattern in *nalC* was G71E. (**c, d**) Gene structure diagrams and genomic qRT-PCR results confirmed that the highly expressed genes did not exhibit CNVs. (**e**) Compared to the OprD protein from imipenem-resistant PA with a well-characterized mechanism in NCBI, our samples did not exhibit the insertion sequence present in the reference sequence. (**f, g**) The relationship between MexAB-OprM and AmpC enzyme overexpression and the expression of their corresponding repressor factors in HR-PA.

In order to further explore the underlying mechanism for the aberrant expression of drug-resistant genes in HR strains, we analyzed the WGS data. Genetic structure analysis revealed the absence of copy number variations (CNVs) in the highly expressed genes (Fig. 5c and d). This finding is consistent with the aforementioned macroscopic analysis of chromosomal collinearity. We identified point mutations in the key regulatory genes *ampR* and *nalC*, respectively; however, these mutations were present in the resistant and non-resistant subpopulations (Fig. 5a and b). Also, it is reported that *oprD* mutations could induce the downregulation of this gene. Therefore, we also conducted a comparative analysis of *oprD* sequences from the 44-M-H and 44-I-H strains against the NCBI database. It showed a high sequence similarity between validated imipenem-resistant PA sequences; however, no insertion sequences (ISs) and mutations were identified (Fig. 5e). Combined with the above results, these findings indicate that the abnormal expression of resistant genes does not result from the genetic defects of these genes.

By conducting the most comprehensive genomic analysis possible, we failed to identify any suspicious mutations associated with HR resulting from dysregulation of MexAB-OprM, AmpC, and OprD expression. Therefore, we hypothesize that the abnormal expression of these genes in heteroresistant strains is correlated with the expression of their regulatory factors. It has been documented that the expression of the efflux pump MexAB-OprM can be influenced by the expression of the upstream repressor *mexR*, which can be inhibited by *armR* (15). Furthermore, the expression of the AmpC enzyme can also be affected by the expression of the repressor *ampR* (16). To further verify whether the overexpression of the MexAB-OprM and AmpC enzymes in HR-PA is associated with dysregulation of repressor expression, we analyzed the relative expression levels of three repressors—*armR*, *mexR*, and *ampR*—in resistant and non-resistant subpopulations. The results showed that the expression regulation of the MexAB-OprM exhibited aberrant repressor trends in 32-HR-ATM, 32-HR-MEM, 44-HR-MEM, 74-HR-ATM, and 74-HR-MEM, primarily manifested as simultaneous overexpression of *mexA* and the repressor *mexR*. In 79-HR-MEM, the expression trends of *armR*, *mexR*, and *mexA* were consistent with the regulatory relationships (Fig. 5f). Regarding the expression regulation of the AmpC enzyme, the repression trends of *ampR* and AmpC were disrupted in 32-HR-ATM, 79-HR-MEM, and 79-HR-ATM isolates, primarily manifested as simultaneous overexpression of AmpC and the repressor factor *ampR*. No statistically significant expression differences were observed between the repressor factor resistance subgroups and non-resistant subgroups in 32-HR-MEM, 44-HR-ATM, 44-HR-MEM, 52-HR-ATM, 74-HR-ATM, 74-HR-MEM, and 76-HR-ATM isolates (0.5 < relative gene expression level < 2, Fig. 5g).

Reports suggest that quorum sensing (QS) systems may be involved in HR mediated by abnormal expression of MexAB-OprM and OprD in PA (17). To validate this hypothesis, we selected three major QS transcription factors—*rhlR*, *lasR*, and *mvfR* (Fig. S4). The results demonstrated no statistically significant correlation between differences in QS system expression and the abnormal expression of MexAB-OprM and OprD in HR-PA (Table S6). Biofilm formation can reflect the expression activity of the QS system (18). To further validate the reliability of qRT-PCR expression analysis results for the QS system, we performed crystal violet biofilm assays on 12 colony pairs. The findings revealed that overexpression of the QS system was associated with differences in biofilm formation (Fig. S5).

**Fig 6:**
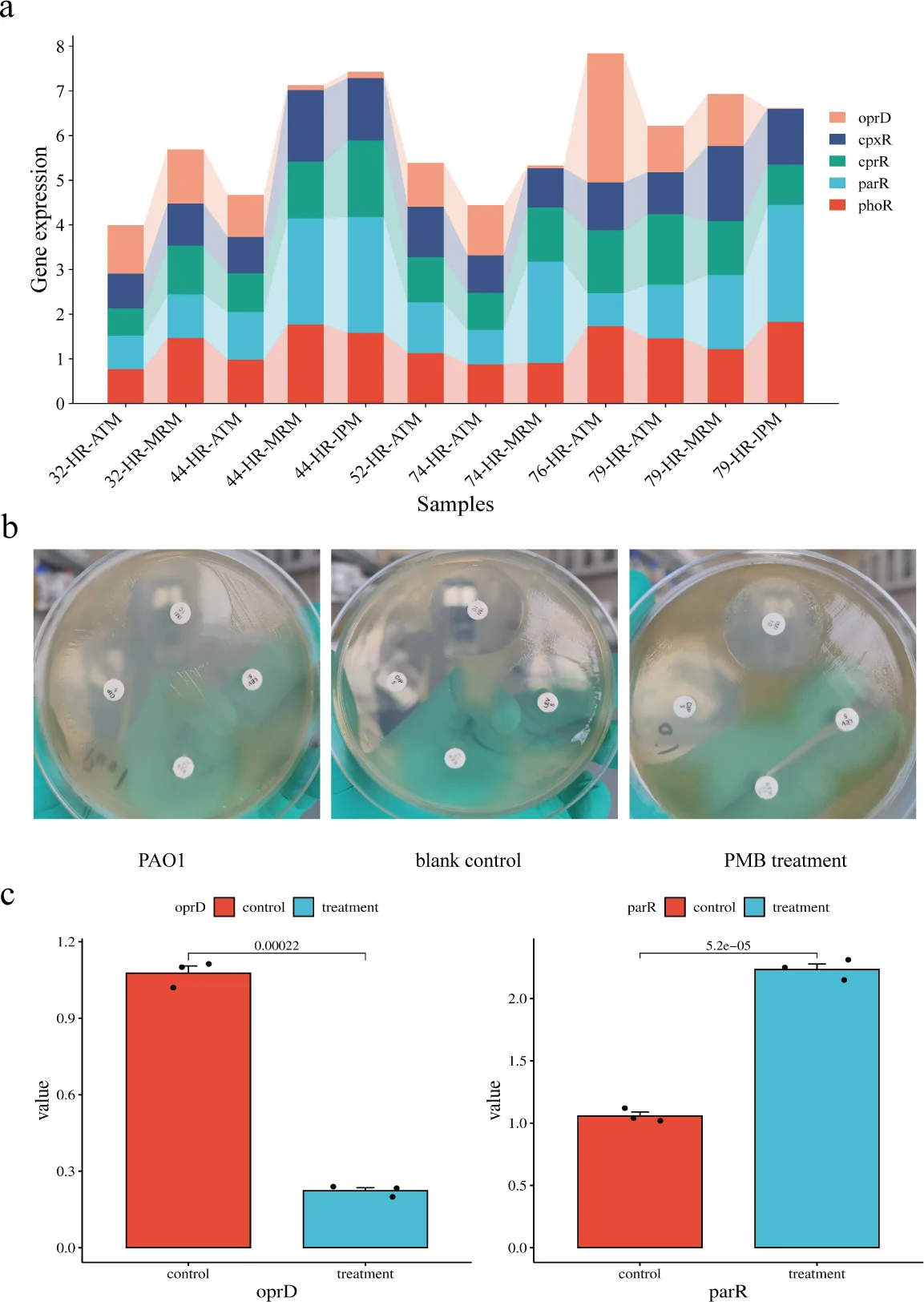
The expression of the TCS ParS/ParR was negatively correlated with the expression of oprD. (**a**) The expression of transcription factor *parR* of TCS was negatively correlated with the expression of *oprD*. (**b, c**) PAO1 induced by PMB exhibited IPM heteroresistance. The qRT-PCR results demonstrated loss of *oprD* expression and overexpression of *parR* in the HR strains (*P* < 0.05).

The two-component system represents a stimulus-response coupling mechanism through which bacteria recognize and respond to complex environmental changes, serving as a potential driver of antibiotic resistance in bacteria (19). Therefore, we hypothesize that resistant subpopulations exist within monoclonal colonies, which may result from rapid regulatory responses of bacterial resistant genes expression under drug stress. To verify this hypothesis, we selected four genes encoding response regulatory proteins of TCS that are explicitly associated with PA antibiotic resistance for correlation analysis (20). Results demonstrated that in the resistant subpopulation, *parR* expression exhibited a negative correlation with *oprD* expression compared to the non-resistant subpopulation (*r* = −0.797, *P* < 0.05. Fig. 6a). However, these genes showed no statistically significant regulatory effect on the expression of *mexA* or *ampC* (*P* > 0.05). It has been reported that antimicrobial peptides such as polymyxin B act as signaling molecules for ParS/ParR (21). To verify whether ParS/ParR regulates *oprD* expression to mediate HR in PA, we conducted HR phenotype validation using PMB-induced PAO1 isolate. The results demonstrated that when the MIC of PMB in Pao1 was induced to 1.0 μg/mL, the K-B test could observe satellite-like colonies in the imipenem inhibition zone (Fig. 6b). Subsequently, we measured the MIC of resistant colonies within the inhibition zone (2.0 μg/mL) and the average MIC of the non-resistant colonies (0.25 μg/mL), revealing an eightfold difference consistent with HR definition. Further analysis using qRT-PCR confirmed the *oprD* and *parR* genes in both resistant and non-resistant subpopulations. The results demonstrated expression loss of the *oprD* gene in resistant subpopulations and overexpression of the transcription factor *parR* gene (Fig. 6c). These data indicated that the two-component system ParS/ParR may play a regulatory role in HR-PA.

## DISCUSSION

Clinical PA infections are typically characterized by prolonged disease course, difficulty in eradication, and elevated drug resistance rates. Although limited clinical reports exist on the impact of HR-PA, its potential clinical hazards should not be overlooked. Bacteria can evade antibiotic pressure through dynamic genomic variations (including low-frequency mutations and transient amplification of resistance genes), leading to HR phenomena in certain bacterial clonal populations (8, 22, 23). Recent retrospective studies have demonstrated that bacterial HR may result in misinterpretation of standard antimicrobial susceptibility testing (AST) results, thereby causing clinical treatment failures, distortion of antimicrobial resistance epidemiological data, and infection recurrence (24). Our study findings indicate that HR-PA is highly prevalent among clinically isolated PA strains at a tertiary teaching hospital in Ningxia, with a detection rate as high as 55.26% (21/38). Among these, MEM, IPM, ATM, LVX, and AMK are the predominant resistant drugs. For HR-PA, conventional clinical AST methods (such as VITEK 2) can only detect approximately 7.27% of HR-PA isolates. This suggests the existence of undetected resistant subpopulations in clinical practice, and HR in PA may pose potential threats to the accuracy of clinical testing and therapeutic outcomes. In fact, significant technological advancements have been made in detecting the specific resistance phenotype of HR, such as microdroplet technology and droplet digital PCR (25, 26). However, the true challenge lies in determining whether patients are infected with heteroresistant strains. Clinicians rely on regional epidemiological survey data to assess the antimicrobial resistance profiles of suspected cases and subsequently request relevant testing from clinical laboratories. Yet, no established clinical standards currently define standardized detection protocols or epidemiological investigation methods for bacterial HR. Furthermore, the scarcity of frontline clinical case reports fails to raise awareness among community physicians about heteroresistant bacteria, who serve as a critical line of defense against antimicrobial resistance outbreaks. Therefore, we emphasize that bacterial HR urgently requires attention from clinical standard-setting authorities to curb its spread.

Currently, there is no standardized method for clinical detection of bacterial HR (2). Different strains or laboratories may employ slightly varying detection protocols. In studies on HR phenotypes, initial screening typically uses the diffusion methods such as the K-B test and E-test, while confirmatory testing predominantly employs PAP (9). PAP serves as one of the methods for identifying methicillin-resistant *Staphylococcus aureus* (MRSA) phenotypes (27). The MRSA exhibits HR characteristics, with cells demonstrating varying resistance levels within monoclonal colonies and resistance clone frequencies ranging from 10^−7^ to 10^−4^. The PAP enables both the determination of MIC values for resistant clones and the calculation of their frequency, laying the foundation for its application in MRSA identification. Given that this characteristic closely resembles HR, scientists have developed specific confirmatory detection methods and threshold requirements for resistance mutation frequencies in heterogeneous vancomycin-resistant *Staphylococcus aureus* (HR-VRSA) (28). A recent methodological study suggests that revised population profile-area under the curve (PAP-AUC) may guide clinical identification and treatment of HR infections (29). However, due to its operational complexity and high cost, PAP remains challenging to implement clinically. The broth dilution method is the CLSI-recommended reference method for bacterial MIC testing. This approach is simple and practical, making it suitable for high-throughput screening experiments. Furthermore, studies have demonstrated that PAP exhibits inconsistent results in detecting carbapenemase-mediated HR in Gram-negative bacteria (30). Therefore, this study investigated the application of broth dilution methods for HR detection. The cgMLST demonstrates superior typing accuracy and sensitivity compared to traditional PFGE and MLST typing methods, making it suitable for retrospective microbiological research and epidemiological surveillance (31). Comparative analysis of broth dilution results with PAP results, combined with cgMLST and whole-genome phylogenetic tree analysis, revealed that the error rates of broth dilution methods in HR detection range from approximately 29.09% (16/55) to 33.33% (6/18). These findings indicate that PAP retains irreplaceable value in HR confirmation, likely due to their ability to reflect drug resistance frequencies in test strains—a characteristic feature of HR. It should also be noted that although the broth dilution method exhibits a relatively high false-positive rate due to its dependence on mutation frequency, it remains applicable in confirming HR.

Through comparative genomic studies and chromatin-based core genome sequence analysis, we found that the resistant subpopulations of HR-PA exhibited high concordance with their non-resistant counterparts in terms of resistance gene profiles, gene collinearity, and core gene sequences. By combining resistance phenotype stability experiments with WGS data, we hypothesize that the mechanism driving HR in PA may not involve known genetic mechanisms that mediate bacterial resistance, such as CNVs of resistance genes, plasmids, or transposons. These findings differ from previously reported mechanisms underlying HR in other Gram-negative bacteria. In contrast, our study revealed that the HR phenotypes of PA against carbapenems and aztreonam were closely associated with the differential expression of the *mexA*, *ampC*, and *oprD* genes—these intrinsic resistance genes located on the PA chromosome primarily contribute to the natural resistance of this pathogen (14). Importantly, we observed significant differences in resistance gene expression profiles among resistant subpopulations isolated from single colonies (e.g., the three subpopulations isolated from PA44 and PA79 exhibited distinct resistance spectra and gene expression profiles). This finding suggests that PA may form resistant subpopulations within a single colony by regulating the expression of different resistant genes on chromatin. Moreover, conventional bacterial susceptibility testing methods struggle to detect these subpopulations, thereby compromising the efficacy of combined antibiotic therapy. Subsequently, in our study of resistance gene regulators, we found that the genes regulating the expression of MexAB-OprM and AmpC enzymes exhibited identical single-nucleotide polymorphisms (SNPs) (e.g., the G71E mutation in the *nalC* gene) in both resistant and non-resistant subpopulations; yet their regulatory outcomes were markedly different. Furthermore, qRT-PCR analysis of genes regulating MexAB-OprM and AmpC enzyme expression demonstrated that the observed differences in resistance gene expression within this specific resistance phenotype were independent of known upstream regulatory elements. The causes of this abnormal regulation may include functional loss mutations in the DNA-binding domains of repressor proteins, impaired post-translational modifications, or the dominant role of other activators. In future research, this issue warrants attention, as it provides crucial evidence for the complexity of resistance gene expression regulation in PA, suggesting the potential existence of unknown regulatory pathways. Previous studies have reported that insertion mutations in the *oprD* gene lead to reduced expression levels and confer imipenem resistance in HR-PA strains (32). This study performed sequence alignment of *oprD* sequences from 44-I-H and 44-M-H using NCBI, revealing high sequence identity between these strains and the sequence registered under ID KM492918.1. Researchers have confirmed that a four-base insertion, “CAGT,” occurs after nucleotide position 747 in this sequence, resulting in decreased *oprD* expression levels (33). However, this mutation was not detected in the sequences of the resistant subpopulations analyzed in this study. These findings suggest that drug resistance gene expression in HR-PA may represent a complex process involving multiple upstream regulatory genes, rather than resulting from a single mutation. In conclusion, these findings indicate that HR may serve as a rapid and irreversible survival strategy for PA under antibiotic stress.

The PA exhibits robust resistance to environmental stressors, including disinfectants and antibiotics, demonstrating rapid perception and response to environmental changes. This is closely associated with its efficient QS system and TCS (34, 35). Contrary to the conclusions of Lu and colleagues (17), we did not find a direct relationship between the effects of quorum sensing and HR. Further verification revealed that the expression differences of QS-related transcription factors are significantly associated with biofilm formation. This suggests that QS-related transcription factors may not play a dominant role in the regulation of endogenous drug resistance gene expression in PA. Indeed, since we considered that transcription factors directly act on the target genes, we did not select the signal perception genes of the QS system (e.g., *rhlI* and *lasI*) as Lu and colleagues did. And then, we cannot rule out the potential impact of the genetic background of the study strains on the results. Among candidate TCS, the ParS/ParR emerged as a specific regulatory factor for *oprD* expression (36). This study validated the correlation between its expression and reduced expression of *oprD* in HR-PA through qRT-PCR and simultaneously induced HR to IPM in the PAO1 strain using PMB. These findings establish ParS/ParR as a potential regulatory node for *oprD* expression-mediated HR to IPM in PA. While we observed disruptions in canonical repressor loops involving *mexR* and *ampR*, no consistent pattern emerged across isolates, suggesting that alternative regulatory pathways may be involved.

The HR mechanism described in this study, which is based on abnormal expression of intrinsic resistance genes, is expected to depend on the extensive gene regulatory network of PA, as it manifests through the following features: (i) the sequences of the abnormally expressed resistance genes are consistent between resistant and non-resistant subpopulations; (ii) currently known upstream regulatory elements (e.g., *mexR* and *nalC*) also maintain sequence consistency between resistant and non-resistant subpopulations but exhibit certain differences at the expression level; however, highly expressed repressor factors do not affect the expression of downstream resistance genes; (iii) the resistance subpopulation exhibits best genetic stability, and no currently known resistance-associated genetic mechanisms were detected; and (iv) TCS is involved in HR mediated by the absence of *oprD* expression. Notably, this study was conducted entirely in laboratory settings, with duplicate samples from the same patient source excluded, potentially failing to reflect dynamic resistance phenotypes in the patient’s body. This limitation is common in most HR studies. Meanwhile, we will validate the findings of this study through large-scale cohort studies and high-throughput transcriptomic analyses conducted in a multicenter setting. Furthermore, emerging bacterial resistance genetic mechanisms, such as promoter mutations or epigenetic modifications, which are difficult to detect by conventional omics methods, require consideration. In summary, future validation of our findings will be strengthened through expanded sample cohorts and integration of advanced omics methodologies to confirm generalizability.

### Conclusion

In conclusion, this study advances our understanding of HR in PA by demonstrating that it is a stable, transcriptionally driven phenomenon distinct from the mutation-dependent HR mechanisms described in other pathogens. The identification of the ParS/ParR two-component system as a regulator of *oprD* opens new avenues for therapeutic intervention. Our findings also underscore the urgent need to refine clinical AST practices to detect these clinically significant but easily overlooked resistant subpopulations. Future work should focus on elucidating the upstream signals that trigger and maintain the transcriptional reprogramming underlying HR, as well as exploring whether similar mechanisms operate in other difficult-to-treat pathogens.

## MATERIALS AND METHODS

### Bacterial strains

From October 2023 to April 2024, the 38 non-repetitive clinical preliminary screening samples of PA were collected from the Medical Experimental Center of the General Hospital of Ningxia Medical University, China. Five colonies were randomly selected from each sample and streaked onto ordinary nutrient agar plates using the streaking method, then cultured overnight at 37°C. One single colony was picked from the purified culture plate and cultured overnight in LB broth at 37°C with shaking 200 rpm for HR screening. Before the preliminary HR screening experiment, bacterial cultures were identified using MALDI-TOF MS (VITEK MS, BioMérieux, France). Simultaneously, antimicrobial susceptibility testing was performed using VITEK 2 Compact (BioMérieux, France), and the results were used to evaluate the impact of HR on clinical AST results (37). The K-B test was used to detect the presence of HR subpopulations in 190 isolates against nine antibiotics (imipenem [IPM], meropenem [MEM], aztreonam [ATM], tobramycin [TOB], ciprofloxacin, [CIP], polymyxin B [PMB], levofloxacin [LVX], amikacin [AMK], and ceftazidime-avibactam [CAZ]). Owing to the low copy number of resistant subpopulations within the group, and to avoid missed detections, we adopted the method proposed by Nicoloff, H. and colleagues (9), and the final concentration of the diluted bacterial suspension was set to 2.0 McF units. A preliminary positive result was indicated when satellite colonies appeared within the zone of inhibition. These satellite colonies were collected near the antibiotic disc and cultured overnight in LB broth at 37°C and 200 rpm for confirmation.

### Population analysis profiling (PAP) and verification of the genetic stability of resistant phenotypes

For the confirmation experiments of HR-PA, we employed population analysis profiling, with specific procedures following the operational methods described by Edgar X. Sherman and colleagues (38). In brief, initially screened positive strains were inoculated into LB broth for overnight culture to enhance bacterial growth. The overnight-cultured bacterial suspension was then subjected to 10× serial dilutions in PBS. To minimize the impact of inoculum effects on results, diluted bacterial suspensions at concentrations of 10^−4^ to 10^−7^ were inoculated onto agar plates containing drug concentrations corresponding to the serial dilution ratios (0.12–64 μg/mL). Finally, after incubation at 37°C for 48 h, colony counts were determined on each plate.

The detection method for the genetic stability of bacterial HR phenotypes is as follows: The resistant subpopulation is passaged five times on LB agar plates. The fifth passage plate is then used to verify resistance changes using the K-B test, and the diameter of the inhibition zone is recorded. The above operations are performed for 10 rounds, resulting in a total of 50 generations.

### Broth microdilution method

The resistance subgroups screened by the K-B method were subjected to MIC determination using the broth microdilution method in accordance with the CLSI M100-2025 guidelines operating standards. Concurrently, parallel tests were conducted on parental strains derived from the resistance subgroups to determine the population average minimum MIC value. The broth microdilution susceptibility plates used in the experiment were provided by Fosun Diagnostics (Shanghai). The experiment was repeated three times in parallel. Based on the definition of HR, bacterial suspensions were pipetted from the wells corresponding to the highest MIC values of positive samples on the microdilution broth susceptibility plates and the lowest MIC values of the corresponding parental strains, then inoculated into LB broth for overnight enrichment culture prior to subsequent molecular biology testing. Finally, samples were classified into resistance subgroups (Group H) and non-resistant subgroups (Group L) according to MIC results.

### Whole-genome sequencing and bioinformatics

The samples exhibiting HR to meropenem, imipenem, and aztreonam were selected for enrichment culture (overnight incubation at 35°C with shaking at 200 rpm in 5 mL of LB broth). After the cultures were centrifuged (10,000 rpm for 10 min), the pellets were collected and then subjected to PacBio and Illumina whole-genome sequencing by Zhejiang Tianke Company.

The cgMLST is performed using the schema established by Hauke Tönnies and colleagues (39). The relevant schema was downloaded from the cgMLST server (https://www.cgMLST.org, last updated in January 2025). To increase the accuracy of cgMLST analysis and validate the typing scheme’s analytical performance in bacterial HR, we concurrently employed whole-genome phylogenetic trees for homology analysis of our selected resistant and non-resistant subgroups. The ChewBBACA local platform predicts cgMLST typing on the basis of the schema. The MSTgold was used to generate the minimum spanning tree from the typing results, and Cytoscape software was used to visualize the minimum spanning tree. Resistance gene prediction was conducted using BLAST software, which compares the amino acid sequences of the target species against the CARD database to obtain functional annotations. Protein interaction network prediction uses the NCBI prokaryotic annotation pipeline for genomic annotation, resulting in annotated SQN files. Protein sequences are extracted from these SQN files and compared to the KEGG protein database (*E*-value < 0.00001) and the Nt nucleotide database (*E*-value < 0.00001) using BLAST software to identify the most similar proteins and obtain functional annotations. Copy number variation analysis (CNV) and insertion sequence (IS) prediction are performed using CNVKIT and ISFINDER, respectively. The prediction of resistant genes transposons was performed using the online analysis platform VRprofile2.

### Crystal violet method for biofilm detection

We referred to the method described by Matysik and colleagues (40) to detect biofilm formation among different subpopulations. Briefly, we first cultured the confirmed highly tolerant and low-tolerant subpopulations overnight on LB agar. The overnight cultures were adjusted to a 0.5 McFarland standard suspension using PBS, diluted 100-fold with TSB broth, and then inoculated into 96-well plates. The plates were incubated at 37°C for 24 h. Each of the above procedures was performed three times in parallel for each strain as biological replicates. After 24 h of incubation, washing and staining were carried out, and the absorbance at a wavelength of 590 nm was measured.

### Polymerase chain reaction (PCR) and quantitative real-time PCR (qRT-PCR)

According to the relevant instructions, high-tolerance group bacterial genomic DNA was extracted using the EasyPure Genomic DNA Kit (Transgen, Beijing, China). The purity of the extracted genomic DNA was analyzed using a NanoDrop 2000/2000C (Thermo Fisher, USA), followed by dilution and quantification. The OXA-50 gene from the genome was amplified using Premix Taq (TaKaRa Taq Version 2.0 plus dye, TaKaRa, Japan). The amplification products were subjected to electrophoresis using a Wide Mini-Sub Cell GT Cell (Bio-Rad, USA) and imaged using a Doc XR (Bio-Rad, USA). According to the manufacturer’s instructions, total RNA from high-tolerance and low-tolerance groups of bacteria was extracted using the TransZol Up Plus RNA Kit (Transgen, Beijing, China). The purity of the extracted total RNA was analyzed using a NanoDrop 2000/2000C (Thermo Fisher, USA), followed by dilution and quantification. Total RNA was reverse transcribed, and genomic DNA was removed using the PrimeScript RT reagent Kit with gDNA Eraser (TaKaRa, Japan). Reverse transcription products were used with 16S rRNA as the reference gene, and qRT-PCR was performed using TB Green Premix Ex Taq II (TaKaRa, Japan). The genomic qRT-PCR experimental method refers to the method of Nicoloff H to verify the prediction results of multi-copy number variations of drug-resistant genes (9). The change in gene expression of ≥2-fold increase or ≤0.5-fold decrease was considered significant. The methods for primer design and specificity verification were referenced from the research of Alicia Rodríguez et al. and Stephen A. Bustin et al. (41, 42), and all primers and reaction conditions are detailed in the supplementary document (Table S7). For each bacterial strain, we established three parallel test wells as biological replicates. The targeted gene expression heatmap data were derived from the mean relative expression values across all three replicate wells.

### The role of the efflux pump MexAB-OprM and β-lactamase AmpC in HR-PA

We prepared MH agar containing avibactam according to the CZA concentration specified in the CLSI M100-2025 guideline. The procedure was as follows: MH agar was prepared according to the manufacturer’s instructions, sterilized by autoclaving at high temperature and pressure, and then cooled to 55°C. Avibactam (20 mg; Aladdin, China) was weighed and dissolved in 1 mL of DMSO by shaking. The resulting avibactam solution was added to the cooled MH agar and mixed thoroughly to obtain MH agar containing avibactam at a final concentration of 4 μg/mL. The CCCP can enhance bacterial sensitivity to antibiotics by inhibiting the function of bacterial efflux pumps (43). We prepared MH agar with a final concentration of 25 µg/mL CCCP and MH agar containing both 4 µg/mL avibactam and 25 µg/mL CCCP, following the aforementioned procedures (44). For the phenotypic validation, we employed the K-B test, as this approach allows direct observation of the response of resistant subpopulations within the inhibition zone under the inhibitor’s effect. The specific experimental procedures are described in the previous HR-PA screening experiment.

### eCIM/mCIM Detection of *Pseudomonas aeruginosa* producing metal β-lactamase

To verify the WGS results and demonstrate that the carbapenem resistance in our studied strain is non-enzyme-mediated, we employed eCIM/mCIM to randomly test the carbapenem-resistant subpopulation included in the study. We randomly selected two strains belonging to the carbapenem-resistant subpopulation and used one metalloenzyme-mediated carbapenem-resistant *Pseudomonas aeruginosa* strain as a control. The specific methods and evaluation criteria are as described in the literature (45).

### Two-component system induction experiment

Firstly, the CLSI M100-2025 guideline was followed to determine the MIC of PAO1 against PMB using the paper disc elution method. The PAO1 strain was preserved at the Medical Experimental Center of the General Hospital of Ningxia Medical University. Next, PAO1 was inoculated into LB broth medium containing a drug concentration slightly below the MIC, with daily replacement of the drug-containing LB broth medium, followed by measurement of the MIC of PMB. When a multiple change in MIC value occurred, the K-B test susceptibility test was performed. The selected drugs included CIP, LVX, MEM, and IPM. Finally, the presence of satellite-like colonies within the inhibition zone of the K-B test susceptibility test was observed, and the MIC value of PAO1 for PMB corresponding to the appearance of satellite-like colonies was recorded. To ensure the reproducibility of this experiment, we conducted parallel experiments using the aforementioned procedures on three monoclonal PAO1 strains, and three sets of biological replicates were established for each monoclonal PAO1 strain. For strains exhibiting satellite-like colonies within the inhibition zones, we performed PAP tests with corresponding antibiotics to confirm whether they represented heteroresistant strains. Finally, the resistant subpopulation with the highest MIC value was selected for subsequent qRT-PCR validation of the target gene.

### Statistical analysis

Correlation analysis of gene expression levels was performed using Spearman’s rank correlation coefficient. Differences in the biofilm formation and expression of each gene of interest were tested using the *t*-test. The correlation between the six drug-resistant genes included in the study and phenotypes was analyzed using the unpaired Wilcoxon test. A *P* value of <0.05 was considered statistically significant. Heatmaps and other statistical graphs were plotted using https://www.bioinformatics.com.cn (last accessed on 10 December 2024), an online platform for data analysis and visualization (46).

## Data Availability

The data sets supporting the conclusions of this article are available in the NCBI repository under the unique persistent identifier and hyperlink to data sets in https://www.ncbi.nlm.nih.gov/bioproject/PRJNA1246568/.

## SUPPLEMENTAL MATERIAL

Supplemental material msystems.00538-26-s0001.pdf Tables S1, S3, and S5 to S7; Fig. S1 to S5.

Supplemental tables msystems.00538-26-s0002.xlsx Tables S2 and S4.

ASM does not own the copyrights to Supplemental Material that may be linked to, or accessed through, an article. The authors have granted ASM a non-exclusive, world-wide license to publish the Supplemental Material files. Please contact the corresponding author directly for reuse.
