# Supplementary material for: Transcriptional regulation drives heteroresistance in Pseudomonas aeruginosa: evidence from genomic and targeted gene expression analysis
Source: mSystems. 2026 Aug 18;11(9):e00538-26. doi: 10.1128/msystems.00538-26 (PMC13595920; doi:10.1128/msystems.00538-26)
Supplement: Supplementary file 1 [file msystems.00538-26-s0001.pdf]

Supplemental Information

Transcriptional Regulation Drives Heteroresistance in *Pseudomonas aeruginosa*: Evidence from Genomic and Transcriptomic Analysis

Running title: Heteroresistance Mechanisms in *Pseudomonas aeruginosa*

Weijie Feng [a], Jia Hou [b], Gang Li [c], He Yin [c], Tiantian Dang [c], Guangqi Li [c], Jiaming Li [c], Min Li [f], Chunhua Song [d]#, Sean X. Leng [e]#, Zhijun Zhao [f,g]#

a General Hospital of Ningxia Medical University, Yinchuan, Ningxia, China.

b Department of Respiratory and Critical Care Medicine, General Hospital of Ningxia Medical University, Yinchuan, Ningxia, China.

c Medical Laboratory Center, General Hospital of Ningxia Medical University, Yinchuan, Ningxia, China.

d Division of Hematology, The Ohio State University Wexner Medical Center, the James Cancer Hospital, Columbus, OH43210, USA.

e Johns Hopkins Center on Aging and Immune Remodeling, Division of Geriatric Medicine and Gerontology, Department of Medicine, Johns Hopkins University School of Medicine and Bloomberg School of Public Health, Baltimore, MD, USA.

f Central Laboratory, Peking University First Hospital Ningxia Women and Children's Hospital (Ningxia Hui Autonomous Region Maternal and Child Health Hospital), Yinchuan, Ningxia, China.

g Third Clinical Medical College, Ningxia Medical University, Yinchuan, Ningxia, China.

#Address correspondence to Zhijun Zhao, Chunhua Song and Sean Leng. Zhijun Zhao's email address is: z15815z@163.com; Chunhua Song's email address is: chunhua.song@osumc.edu; Sean Leng 's email address is: sleng1@jhu.edu

Weijie Feng, Qian Zhang and Jia Hou contributed equally to this work. The order of authors is determined based on their contribution level.

Supplemental Tables

Supplemental Table.1 Comparison of PAP results with those obtained by broth microdilution method.

Drug / Sample	PAP							BMD						
	MRM	IPM	LEV	TO	ATM	AK	CZA	MRM	IPM	LEV	TO	ATM	AK	CZA
2	8/0.5	16/0.5	16/0.5					8/0.5	8/0.5	8/0.25				
8	8/0.25		4/0.5			16/1		8/0.25		4/0.5		16/1	8/1	
14				2/0.25		8/0.5		4/0.25	4/0.5		2/0.25		8/0.5	
15						16/1		4/0.5	8/0.5				16/1	
32	8/0.5		16/0.5		≥64/8	16/1		8/0.5	8/1	16/0.5		128/8	8/1	
35	4/0.5	8/0.5				1/0.12		4/0.5	8/0.5				2/0.25	
43					32/0.5				4/0.25	2/0.12		32/0.5		
44	16/1	16/2			32/0.5			8/1	16/1			16/0.5		
47		32/2						16/1	16/2					
50	≥64/8	≥64/8				8/0.5	≥64/2	64/8	64/8				8/0.5	128/2
52			2/0.25		16/2			4/0.25	32/0.5	2/0.25		16/2		
53	32/0.5		8/0.5					32/0.5		8/0.5				
54	16/1	32/1	4/0.5		16/2			16/1	16/1	4/0.25		16/2		
74	16/0.5				16/1		4/0.25	16/1	32/1		2/0.25	16/1		4/0.25
75	≥64/1		2/0.12					128/1		2/0.25			2/0.25	
76			4/0.25		32/1			2/0.25	4/0.5	4/0.25		32/1		
79	32/0.5	8/1			32/2			16/0.5	8/1			32/2		
86	64/0.5	16/0.5	2/0.25		≥64/4			16/0.5	16/0.5	2/0.25		128/4		
88	4/0.5				≥64/2	2/0.25		4/0.5				256/4	2/0.25	
89		32/1				8/1			64/1				8/1	
102	8/0.25		1/0.12		4/0.5			8/0.5		2/0.25		4/0.5		

Note: Values indicate resistance subpopulation MIC/non-resistance subpopulation MIC. The red font indicates strains detected by the broth microdilution method that meet HR criteria, while those undetected by the PAP. PAP, population analysis profile; BMD, broth microdilution method.

Supplemental Table.3 Assess results of genetic stability of resistance phenotypes in HR-PA.

Day / Sample	0day	5day	10day	15day	20day	25day	30day	35day	40day	45day	50day
32-HR-ATM	10mm	10mm	11mm	10mm	11mm	9mm	10mm	9mm	11mm	9mm	12mm
32-HR-MRM	9mm	11mm	10mm	10mm	11mm	9mm	10mm	9mm	11mm	10mm	11mm
44-HR-ATM	9mm	10mm	10mm	9mm	10mm	10mm	11mm	10mm	10mm	9mm	12mm
44-HR-MRM	10mm	10mm	9mm	10mm	9mm	11mm	11mm	11mm	12mm	9mm	10mm
44-HR-IPM	10mm	11mm	10mm	10mm	11mm	12mm	10mm	12mm	9mm	10mm	9mm
52-HR-ATM	10mm	11mm	10mm	9mm	9mm	10mm	11mm	10mm	12mm	11mm	9mm
74-HR-ATM	11mm	10mm	10mm	10mm	9mm	10mm	9mm	11mm	10mm	12mm	11mm
74-HR-MRM	10mm	10mm	9mm	11mm	9mm	10mm	12mm	10mm	11mm	11mm	12mm
76-HR-ATM	9mm	11mm	10mm	11mm	10mm	11mm	10mm	9mm	12mm	10mm	12mm
79-HR-ATM	10mm	10mm	11mm	10mm	9mm	11mm	10mm	10mm	10mm	9mm	10mm
79-HR-MRM	11mm	10mm	10mm	10mm	10mm	10mm	11mm	11mm	11mm	10mm	12mm
79-HR-IPM	10mm	11mm	10mm	11mm	11mm	10mm	9mm	10mm	11mm	10mm	11mm

Note: All data in the table represent the actual measured inhibition zone diameters. A difference of more than 3 mm between two consecutive measurements is considered indicative of a change in antimicrobial susceptibility phenotype.

Supplemental Table.5 Validation of transcriptome results.

	MRM	IPM	ATM	AK	CIP
32-ATM	R/S[1]	S/S	R/S	S/S	S/S
32-MRM	R/S	I/S	R/I	S/S	S/S
44-MRM	R/S	R/S	R/I	S/S	S/S
44-IPM	S/S	R/S	S/S	S/S	S/S
44-ATM	S/S	S/S	R/S	S/S	S/S
52-ATM	S/S	S/S	R/S	S/S	S/S
74-MRM	R/S	R/S	R/S	S/S	S/S
74-ATM	S/S	S/S	R/S	S/S	S/S
76-ATM	S/S	S/S	R/S	S/S	S/S
79-MRM	R/S	R/S	R/S	S/S	S/S
79-ATM	S/S	S/S	R/S	S/S	S/S
79-IPM	S/S	R/S	S/S	S/S	S/S

Note: The result evaluation criteria refer to the CLSI M100-2025 guidelines.

Supplemental Table.6 Correlation analysis of QS with efflux pumps, OprD, and AmpC.

	ampC		*mexA*		*oprD*	
	r	*P*-value	r	*P*-value	r	*P*-value
rhlR	0.33	0.291	0.34	0.282	-0.254	0.415
lasR	0.56	0.058	0.53	0.075	-0.454	0.144
mvfR	-0.35	0.264	0.26	0.419	-0.18	0.576

Supplemental Table.7 Primer

Gene	Primer type		Sequence (5'->3')
mexA	qPCR	Forward primer	AAGCGCCTGTTCAAGGAAGG
		Reverse primer	CCTTGGTGTAGCGCAGGTTG
mexC	qPCR	Forward primer	TTGGCTATGGCCATCGCGTT
		Reverse primer	ATCGAAGTCCTGCTGGCTGA
mexE	qPCR	Forward primer	ATCCCACTTCTCCTGGCGCT
		Reverse primer	GGTCGCCTTTCTTCACCAGT
mexX	qPCR	Forward primer	CCGTGCTGTTCCAGATCGAC
		Reverse primer	TCCTTGATCAGGTCGGCGTA
oprD	qPCR	Forward primer	CTAGCCTCCTATGGCGTTCC
		Reverse primer	AGGTTGGTTTCGTGGTGCTT
ampC	qPCR	Forward primer	GGGCTGGCCTCGAAAGAGGAC
		Reverse primer	GCACCGAGTCGGGGAACTGCA
16S	qPCR	Forward primer	GGAGGAAGGTGGGGATGACG
		Reverse primer	ATGGTGTGACGGGCGGTGTG
OXA-50	PCR	Forward primer	TTCCCGCTTCCACCTACAAG
		Reverse primer	CAGAAAGCGGGTCTGTTCCA
lasR	qPCR	Forward primer	CCTGTGGATGCTCAAGGACTAC
		Reverse primer	CCATATGGAAGTTCACATTGGCTTC
rhlR	qPCR	Forward primer	TAGTTCTGCATCTGGTATCGCTC
		Reverse primer	GGCTTCGATTACTACGCCTATGG
mvfR	qPCR	Forward primer	CATGTTGAAATCGTCGAGCAGTAC
		Reverse primer	CGACATCGCCTTCAATCTCAAC
cpxR	qPCR	Forward primer	CAAGCTGACCCTCTACGACC
		Reverse primer	TAAAGACCTTGTGTCGGCGG
phoP	qPCR	Forward primer	CTCTACCGGGTCAGCGAATAC
		Reverse primer	GATCAGGATCGGGAAGGACTTG
parR	qPCR	Forward primer	GATGTTCAGCTTGCCGAATGTC
		Reverse primer	GGACGATCTCGATCACATCCTC
cprR	qPCR	Forward primer	CTGGAAGATCCTTGAGTGCCTG
		Reverse primer	CACGTTGAGGGTGTTGCTTTC
armR	qPCR	Forward primer	CCCGCTCAGTAGAAGTGCTC
		Reverse primer	GAACACTCCGCGCAACAAAC
mexR	qPCR	Forward primer	CGCGTTGTTCGTCGATAAGC
		Reverse primer	AACTACCCCGTGAATCCCGA
ampR	qPCR	Forward primer	TAGCTGCCGGTACTCACTTC
		Reverse primer	GAGCATCGTCTTCGACACCT

Supplemental Figures

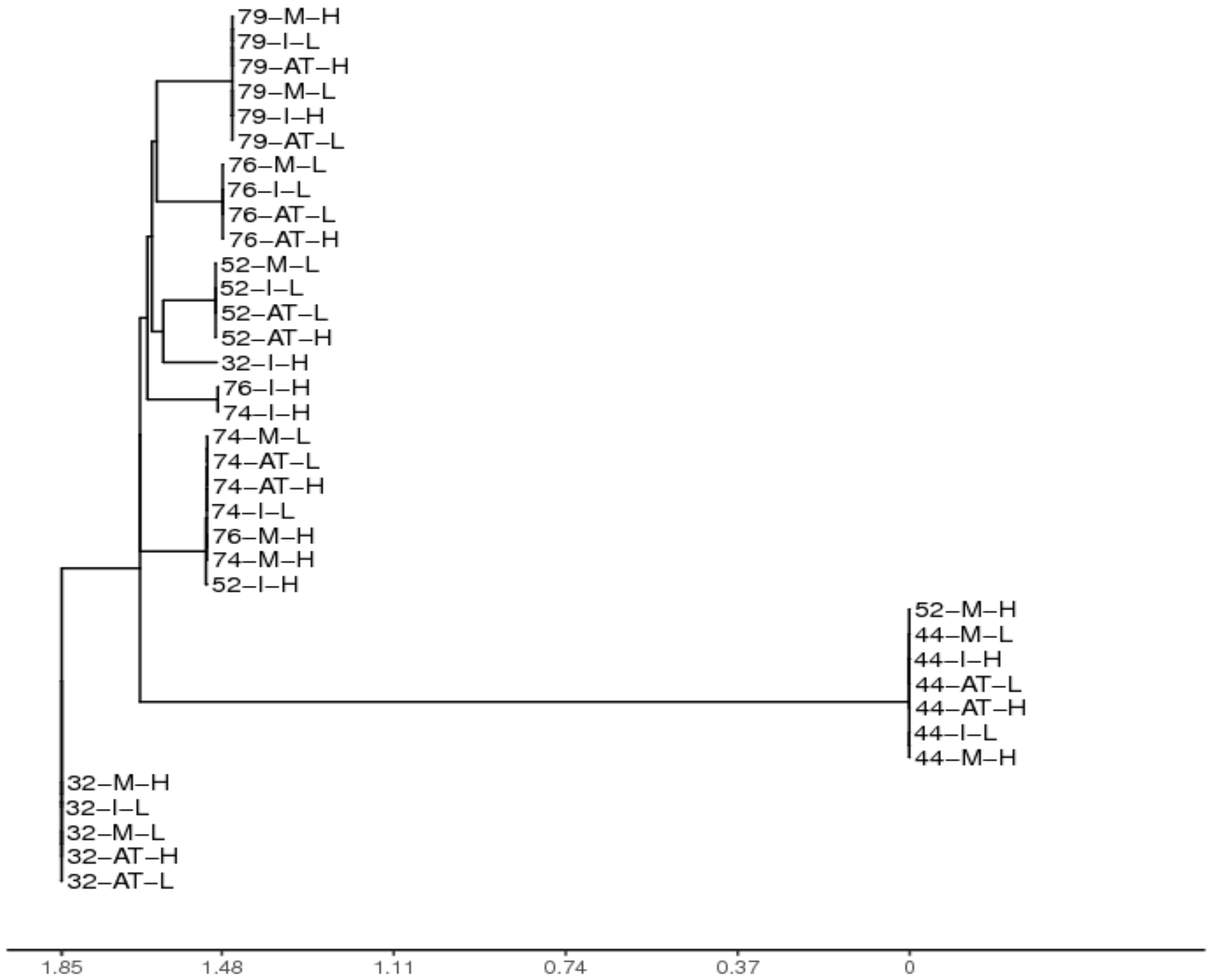

Supplementary Fig. 1 Whole-genome phylogenetic tree

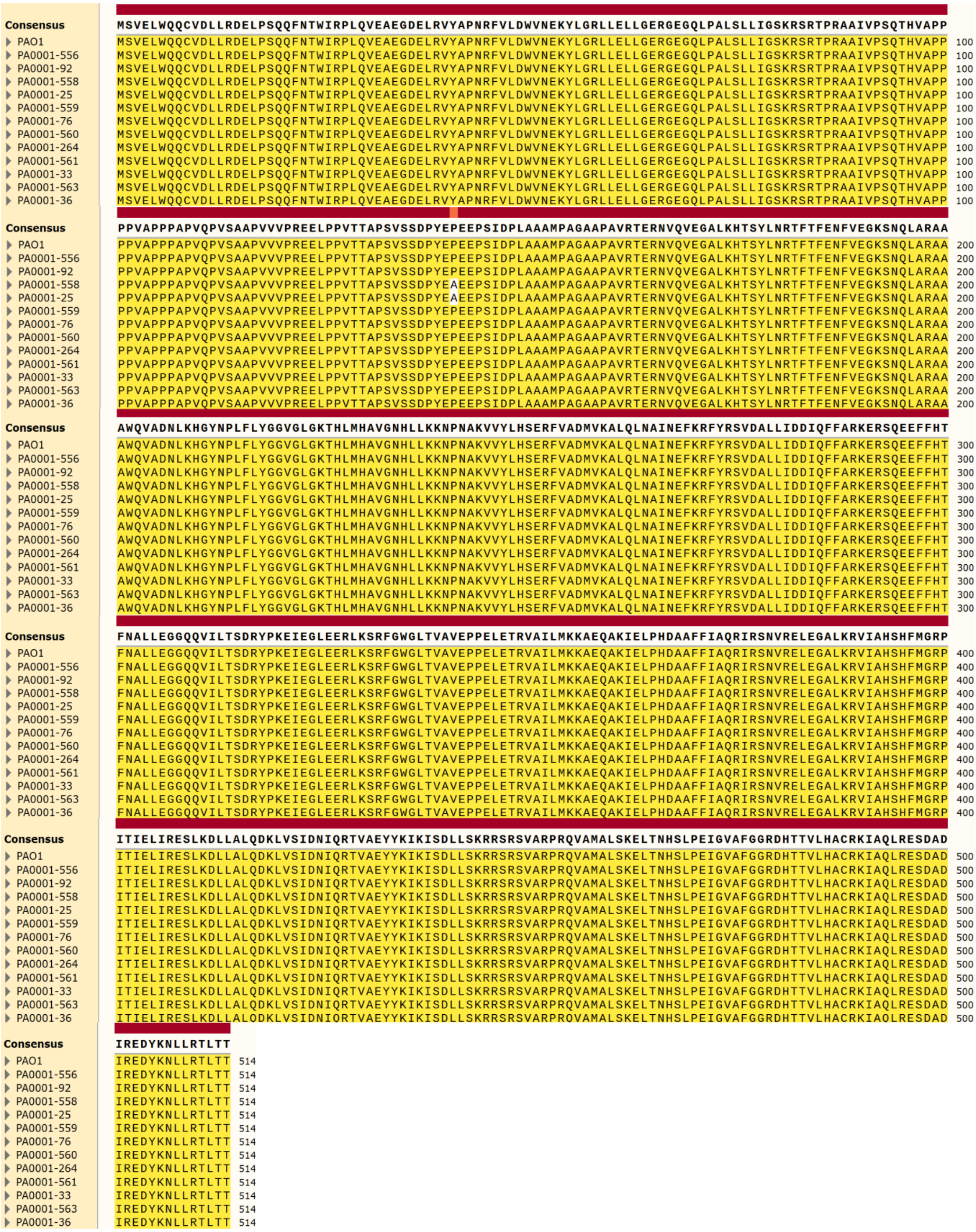

Supplementary Fig. 2 *dnaA* gene amino acid sequence alignment results

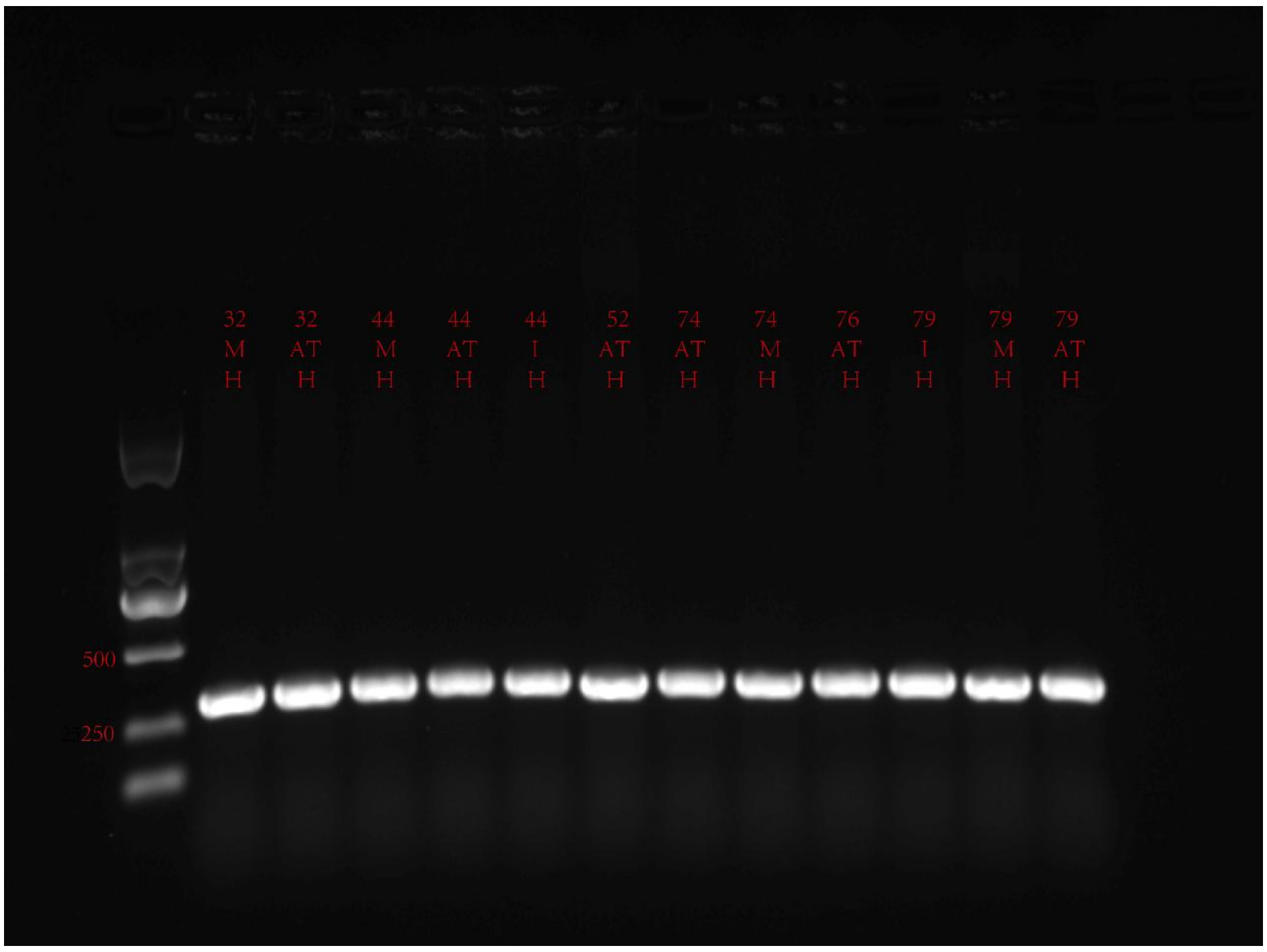

Supplementary Fig. 3 Full-length gels

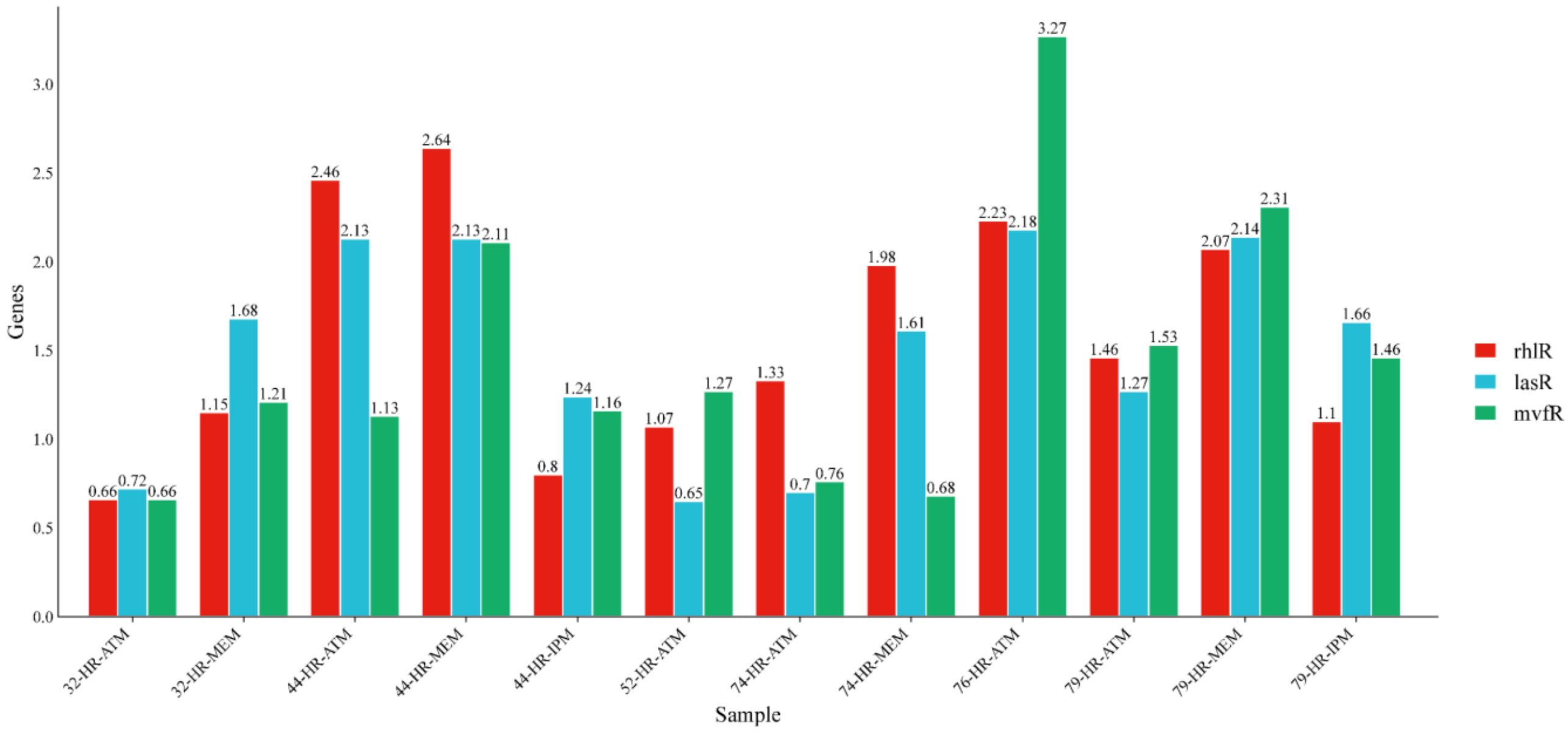

Supplementary Fig. 4 Expression status of QS transcription factors

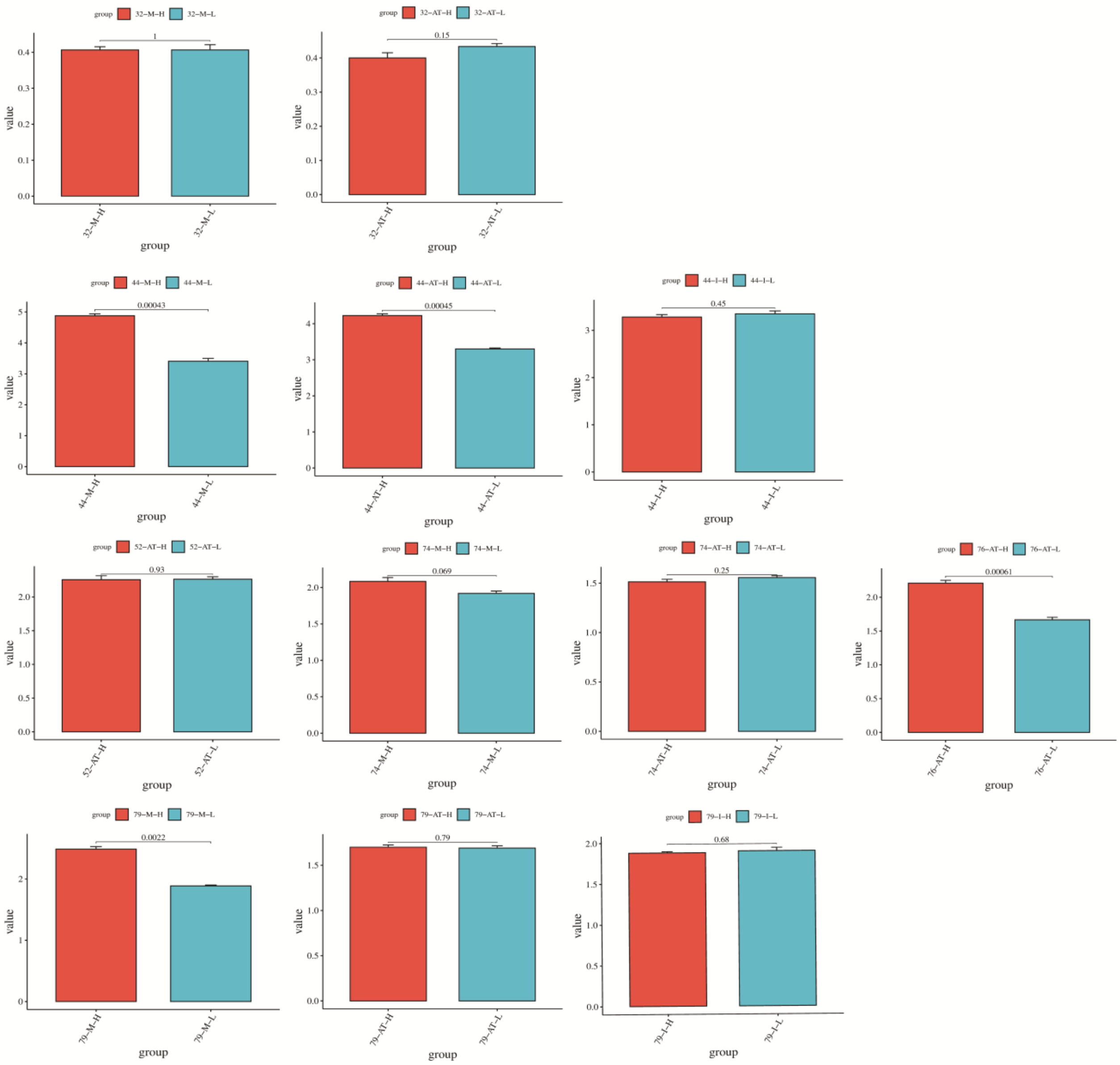

Supplementary Fig. 5 Biofilm determination by crystal violet method. Significant differences in QS system expression were observed among the four sample groups (44-HR-ATM, 44-HR-MRM, 76-HR-ATM, and 79-HR-MRM), with corresponding biofilm staining results showing statistically significant variations ($P<0.05$).
